# Evaluation of Glycosylated Ferritin in Adult-Onset Still’s Disease and Differential Diagnoses

**DOI:** 10.3390/jcm11175012

**Published:** 2022-08-26

**Authors:** Arthur Guerber, Etienne Garneret, Thomas El Jammal, Sabine Zaepfel, Mathieu Gerfaud-Valentin, Pascal Sève, Yvan Jamilloux

**Affiliations:** 1 Internal Medicine, University Hospital Croix-Rousse, Hospices Civils de Lyon, 69004 Lyon, France; 2 Faculty of Medicine, Claude Bernard University Lyon 1, 69622 Villeurbanne, France; 3 Biochemistry Department, University Hospital Croix-Rousse, Hospices Civils de Lyon, 69004 Lyon, France; 4 Research on Healthcare Performance (RESHAPE), INSERM U1290, Université Claude Bernard Lyon 1, 69373 Lyon, France; 5 Lyon Immunopathology Federation (LIFE), 69000 Lyon, France

**Keywords:** glycosylated ferritin, adult-onset Still’s disease, diagnostic accuracy, hemophagocytic lympho-histiocytosis

## Abstract

Glycosylated ferritin (GF) has been reported as a good diagnostic biomarker for adult-onset Still’s disease (AOSD), but only a few studies have validated its performance. We performed a retrospective study of all adult patients with at least one GF measurement over a 2-year period in one hospital laboratory. The diagnosis of AOSD was based on the expert opinion of the treating physician and validated by two independent investigators. Patients’ characteristics, disease activity, and outcome were recorded and compared. Twenty-eight AOSD and 203 controls were identified. Compared to controls, the mean GF was significantly lower (22.3% vs. 39.3, *p* < 0.001) in AOSD patients. GF had a high diagnostic accuracy for AOSD, independent of disease activity or total serum ferritin (AUC: 0.674 to 0.915). The GF optimal cut-off value for AOSD diagnosis was 16%, yielding a specificity of 89% and a sensitivity of 63%. We propose a modified diagnostic score for AOSD, based on Fautrel’s criteria but with a GF threshold of 16% that provides greater specificity and increases the positive predictive value by nearly 5 points. GF is useful for ruling out differential diagnoses and as an appropriate classification criterion for use in AOSD clinical trials.

## 1. Introduction

The diagnosis of adult-onset Still’s disease (AOSD) is challenging because of overlapping features with infections, malignancies, or other rheumatic diseases [1]. Various sets of classification criteria have been published so far, with those by Yamaguchi et al. being the most commonly used [1]. These criteria are not intended for diagnostic purposes. In 2002, Fautrel et al. proposed a revised set for classification that included the level of the glycosylated ferritin (GF) fraction (i.e., ≤20%) as a major criterion [2]. These criteria were further validated by the same team in 2018 [3]. Importantly, GF reduction has been observed in other conditions, such as severe acute respiratory syndrome coronavirus 2 (SARS-CoV-2) infection or hemophagocytic lympho-histiocytosis (HLH) [4,5,6]. It is noteworthy that Still’s disease and HLH are closely related, as 5–19.5% of patients have overt HLH [7,8,9,10,11], and 16.7–53% have bone marrow features of hemo-phagocytosis [12,13,14]. Some authors have even raised the question of a unique continuum between the two diseases.

Despite these findings, GF is still understudied and underutilized, maybe due to a lack of robust validation studies or limited access to its dosage [15]. Unlike GF, total serum ferritin (TSF) has been repeatedly reported as a hallmark of AOSD, mostly for extreme values [16,17,18]. However, TSF lacks sensitivity and specificity [19].

The objectives of this study were therefore to evaluate the diagnostic performance of GF in AOSD and to describe its course in AOSD and its main differential diagnoses.

## 2. Materials and Methods

### 2.1. Study Design and Population

We conducted a retrospective, case-control study by collecting data from all adult patients who had at least one GF measurement between 1 January 2018, and 31 December 2019 (before the COVID-19 pandemics). The exclusion criteria were age under 18 years and/or patient without any management or follow-up in Lyon university hospitals and/or who had incomplete or missing medical records in our center. The Lyon, France institutional biochemistry laboratory, where the assays were performed, receives samples from five French university hospitals. In France, only two public laboratories carry out GF testing. The institutional review board approved the study protocol (#22-862). In accordance with French legislation on non-interventional retrospective studies, no written informed consent was required for inclusion.

### 2.2. Glycosylated Ferritin Assays

The GF fraction was determined using Worwood’s procedure [20], with minor modifications previously described and validated [21]. This method separated ferritin glyco-forms based on their affinity for concanavalin-A (Con-A). Separation was performed on a Con-A-sepharose-4B gel (Amersham-Biosciences^®^, Piscataway, NJ, USA). Then, after a centrifugation step, the Con-A-unbound fraction (unglycosylated) was measured in the supernatant using luminescent oxygen channeling (LOCI) assay (SIEMENS-Dimension^®^-Vista™1500, Munich, Germany). The GF fraction (normal range: 50–80%) was expressed as the percentage of TSF (normal range: 13–200 µg/L). The limit of quantification of the GF measurement with our assay technique was 14%.

### 2.3. Data Collection and Classification

Biological data were automatically extracted from two electronic patient data management systems (GLIMS9-MIPS and EASILY-HCL). Clinical data were collected using a standardized form. The study period was defined as the time between GF measurement and the last available data in the medical record.

Considering the time of GF measurement, three situations were distinguished: (i) during a disease flare (including disease onset), (ii) during disease remission, or (iii) during a disease complication, including HLH. The Hscore was calculated retrospectively for all patients [22]. Disease activity in cases and controls was classified as active (i.e., flare) if clinical or biological signs of the disease were present at the time of GF determination, or non-active (i.e., remission) if not.

The diagnosis of AOSD was based on the clinico-biological presentation, disease course, and management. The Fautrel and Yamaguchi classification criteria were sought to assist in the classification of patients but were not mandatory to retain the diagnosis. The diagnosis was retained if: (i) it was confirmed by the referring physician at the last follow-up, (ii) it was validated by two independent investigators (Y.J. and A.G.), with conflicting cases discussed with a third expert (PS or MGV) and finally classified by consensus. Differential diagnoses (i.e., exclusion criteria of the Yamaguchi set) were always sought and ruled out: infections, especially sepsis and EBV infection; malignant diseases, especially lymphoma; and inflammatory rheumatic diseases. Controls were all the patients who had at least one GF assay during the study period in the same biochemistry laboratory as cases, but who did not meet the predefined AOSD definition. Due to the retrospective nature of the chart review, it was not possible to determine the precise reason why the clinicians who requested the GF test did so, since it is not recorded in the biochemistry laboratory’s records. However, three main reasons seem to have motivated the prescription: etiological workup of unexplained fever or pericarditis, diagnostic suspicion of AOSD in front of evocative symptoms in a patient, and diagnostic support of a suspected HLH.

In the control group, six etiological subgroups were considered: infectious diseases, solid cancers, hematological malignancies, other immune-mediated inflammatory diseases (IMIDs), acute hepatitis, and a group encompassing all other less frequent conditions. The classification of patients into each subgroup was based on the diagnosis made by the treating physician. Each case was reviewed by the two independent investigators (Y.J. and A.G.) and confirmed by a third expert (PS or MGV), if necessary. In case of residual uncertainty, cases were excluded.

### 2.4. Outcomes

Patient outcomes were assessed using a composite prognostic variable that included death or intensive care unit admission within one month of the GF test. Survival time was calculated from the date of the GF test to the time of death or intensive care unit (ICU) transfer. Severity scores (e.g., SOFA score) were not calculable due to the lack of data available at the time of ICU admission.

### 2.5. Statistical Analysis

Continuous variables were described as means (±SD) if they were normally distributed, or as medians (Q1–Q3) if not. Categorical variables were described as percentages. Quantitative variables were compared using a student’s *t*-test if they were normally distributed or using a Mann-Whitney-test if not. Categorical variables were compared using chi-squared test or exact Fisher’s test, as appropriate. Analysis of variance (ANOVA) was used for multigroup comparison with variables of normal distribution, whereas Kruskal-Wallis test was used if the normality assumption was not satisfied. Receiver operating characteristics (ROC) curves were plotted and areas under the curves (AUC) were computed to assess the performance of GF for the diagnosis of AOSD. Specificity (Sp), sensitivity (Se), positive predictive value (PPV), and negative predictive value (NPV) were calculated for different thresholds. The best cut-off values were determined using the Youden index. The best prognostic threshold for GF was calculated with maximally selected rank statistics. Overall survival was estimated using the Kaplan-Meier method. Survival curves were compared using the log-rank test. Multivariate analyses were performed using a Cox regression model. Confidence intervals (CI) were calculated at 95%. For all statistical analyses, *p* < 0.05 was considered significant and all tests were two-tailed. Analyses were performed using the R software v 4.1.2 (R Foundation for Statistical Computing, Vienna, Austria). Figures were performed using GraphPad Prism version 8.0.2 for Windows (GraphPad Software, San Diego, CA, USA)

## 3. Results

### 3.1. Study Population and Comparison

231 patients were included in the analysis, of which 28 (12%) patients met the predefined definition of AOSD, and 203 were categorized as controls. The comparison of the main clinical and biological characteristics between cases and controls is shown in Table 1. Fever, adenopathy, arthralgias, pharyngitis, maculopapular rash and/or transient erythema, hepatic cytolysis predominantly on ALT, and a neutrophilic polynucleosis were more frequent in AOSD than in controls and are the hallmark of the disease. Patients were about ten years younger in AOSD than in controls. Median TSF was not different between AOSD cases and controls (1162 vs. 717 µg/L, *p* = 0.194) unlike mean GF, which was significantly lower in AOSD (22.3 vs. 39.3%, *p* < 0.001). There was no difference in the frequency of HLH, indicating the absence of confounding bias due to this condition for the GF assessment.

The distribution of causes in the control group is shown in Table 2. The three most common diagnostic groups were other IMIDs (35%), infectious diseases (25%), and neoplasia (17%), mainly hematologic malignancies.

**Figure 1:**
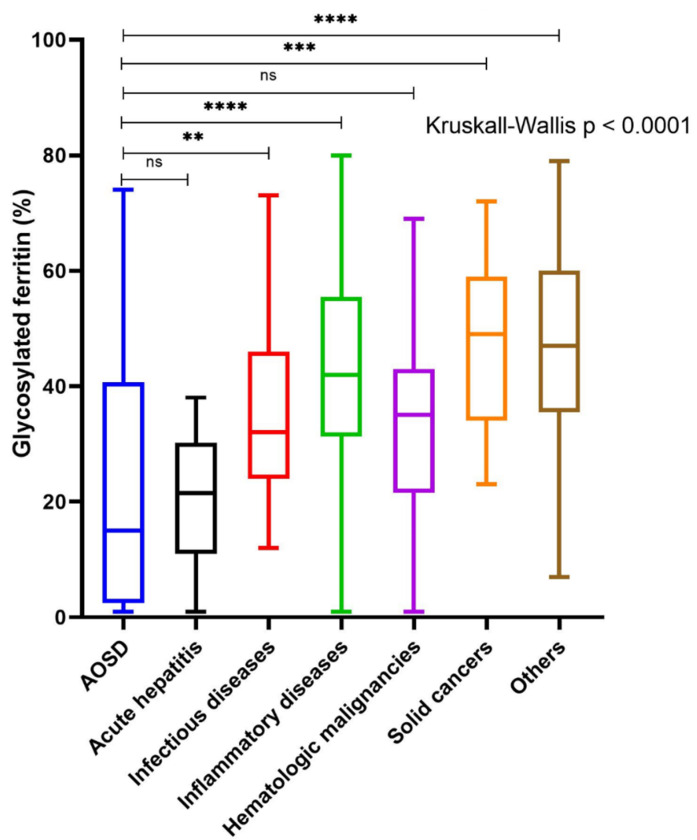
Glycosylated ferritin level between adult-onset Still’s disease (AOSD) and controls according to the different etiological groups. Abbreviations: ** *p* < 0.01; *** *p* < 0.001; **** *p* < 0.0001; ns: not significant.

The median GF level was 15% (4–40) in AOSD patients and was significantly lower than that of patients with infectious diseases (32% (24–46), *p* < 0.01), IMIDs (42% (32–55), *p* < 0.0001), solid cancers (49% (38–57), *p* < 0.001), and with other conditions (50% (37–60), *p* < 0.0001) (Figure 1). Conversely, there was no significant difference in median GF between AOSD patients and those with acute hepatitis (18% (10–30), *p* = 0.88) or hematological malignancies (35% (23–43), *p* = 0.09).

GF had prognostic value when all causes were considered with an optimal cutoff point calculated at 40% (Supplementary Figure S1). Multivariate analysis showed that GF levels were significantly associated with mortality and ICU admission, independent of age, gender and HLH (*p* = 0.04).

### 3.2. Causes According to the Level of Glycosylated Ferritin

**Figure 2:**
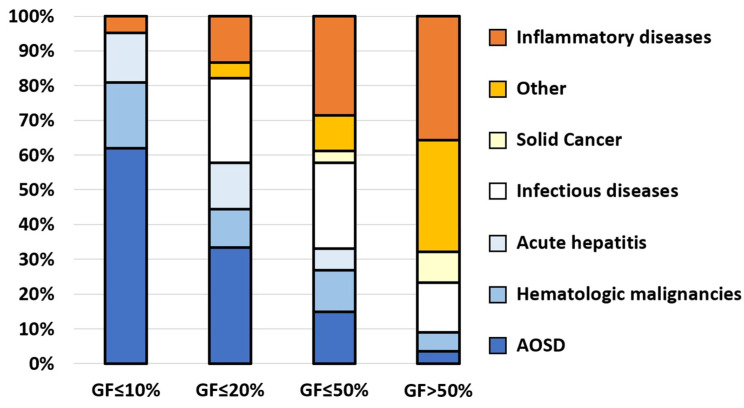
Cause prevalence according to the glycosylated ferritin (GF) level in adult-onset Still’s disease (AOSD) and control subgroups.

The prevalence of the different causes according to the level of GF is presented in Figure 2. Among the 45 patients with a GF level below the previously described threshold of 20% [23,24], the diagnoses were: AOSD in 33%, infections in 25%, acute hepatitis in 13%, IMIDs in 13%, and hematological malignancies in 11%. Of note, 25% (*n* = 11) of these 45 patients had HLH complicating the underlying disease, of which three had AOSD. Therefore, one in two patients (22/45) had neither HLH nor AOSD despite a GF ≤ 20%.

In the infectious disease subgroup, patients with a GF ≤ 20% had mainly viral infections (8/11) while pyogenic bacterial infections were in the minority (3/11). Noteworthy, none of the viral infection with decreased GF were complicated by HLH. In viral infection the median GF was significantly lower than in other infections (26 vs. 34%, *p* = 0.043). In the hematological malignancy subgroup, all patients with a GF ≤ 20% were complicated by HLH, whereas none with GF > 50% was (Supplemental Figure S2). The median GF of hematologic malignancies without HLH was significantly higher than that of AOSD without HLH (43 vs. 24%, *p* = 0.02). The only patient with a GF ≤ 10% in the IMID subgroup had an HLH in the setting of a genetic NLRC4-MAS syndrome. Thus, only three causes were associated with a GF level ≤ 10%: HLH, AOSD, and acute hepatitis. Of note, the median alanine aminotransferase (ALT) level was significantly lower in AOSD (during flare) than in acute hepatitis (84.5 vs. 641 IU/L, *p* < 0.001), suggesting the usefulness of ALT in distinguishing an AOSD flare from unrelated acute hepatitis.

### 3.3. Glycosylated Ferritin Level as a Function of Disease Activity

**Figure 3:**
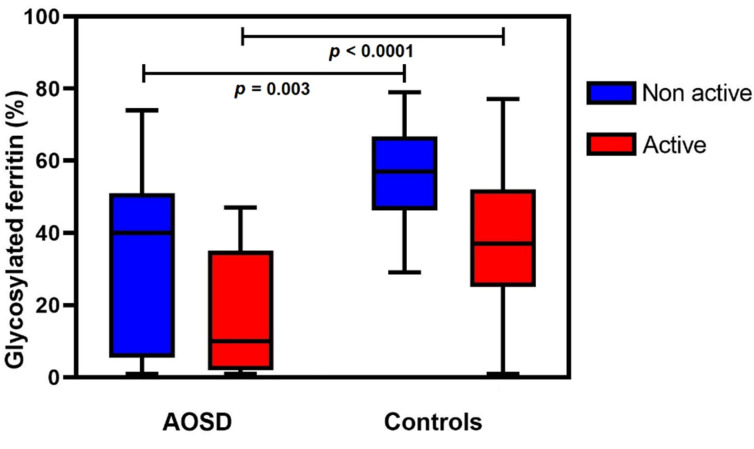
Glycosylated ferritin level according to disease activity in adult-onset Still’s disease (AOSD) and controls.

Mean GF levels according to disease activity were then compared between AOSD patients and controls (Figure 3). GF levels were significantly lower in AOSD patients whether the disease was active (17 vs. 38%, *p* < 0.0001) or not (33 vs. 56%, *p* = 0.003). Patients with AOSD flare had lower mean levels of GF than AOSD patients in remission (17 vs. 33%) but the difference was not statistically significant. Longitudinal data (i.e., different timepoints for the same patient) were not available.

A subsequent analysis of patients with TSF < 1000 µg/L showed that the mean GF levels were also significantly lower in AOSD patients than in controls (33 vs. 46%, *p* = 0.004) showing the diagnostic value of the marker independently of the ferritin level. Finally, there was no relevant statistical correlation between GF levels and other clinical or biological variables, in particular TSF (Pearson’s r = −0.28, *p* < 0.0001) and fibrinogen levels (Pearson’s r = 0.11, ns).

### 3.4. Glycosylated Ferritin Performance for the Identification of AOSD

**Figure 4:**
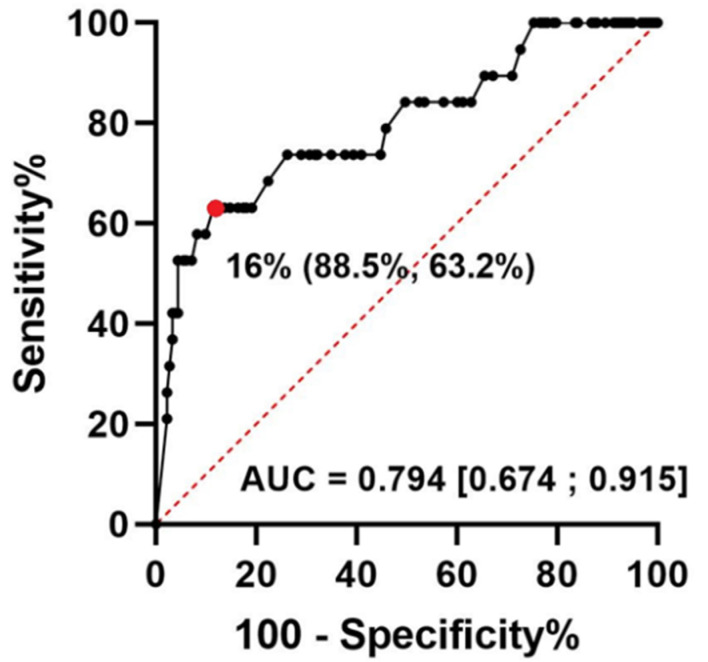
Receiver operating characteristics curve for glycosylated ferritin in adult-onset Still’s disease flare. Abbreviations: AUC, area under curve; (specificity %, sensibility %); (Confidence intervals at 95%). The red dot is the best threshold value determined using the Youden index.

GF had a good accuracy for the diagnosis of AOSD, with an AUC of 0.794 (95% CI, 0.674–0.915, Figure 4). The optimal cut-off was 16% and yielded a Sp of 88.5%, Se of 63.2%, NPV of 95.9%, and PPV of 36.4%. 

Several GF values and different diagnostic sets were then considered to assess their accuracy for active AOSD (Table 3). As expected, a decreasing threshold led to better specificity and less sensitivity. In contrast, a GF threshold ≤ 50% had a sensitivity and NPV of 100%. We further analysed GF combination with various sets of criteria. In our series, Yamaguchi classification (not including GF) had high PPV and NPV and was slightly more accurate than Fautrel’s, which include GF as a major criterion. Only one patient with DRESS syndrome was wrongly classified as positive by the Yamaguchi classification compared to six false positives with the Fautrel criteria. To gain insight into the performance of the GF in combination with the previously described classification, we modified the Fautrel criteria by changing the GF threshold to 16%. The modified score (tentatively called Fautrel-16) reduced the number of false positives, improved the specificity of the criteria without affecting their sensitivity, and increased the PPV from 68.4% at the 20% threshold to 72.2% at the 16% threshold.

## 4. Discussion

This study validated the importance of GF as a biomarker for AOSD diagnosis. Despite the strong and independent statistical association between the decrease in GF level and the diagnosis of AOSD, our study suggested that the previously reported threshold of 20% may lack specificity if considered alone. Indeed, two-thirds of the patients had a diagnosis other than AOSD at this level, whereas this proportion was only half in the original series [24]. The main reason for such differences was possibly the etiological distribution in the control groups. Though unselected, the control group from the original cohort was composed of a greater proportion of patients with IMIDs (52 vs. 35%) but lower proportions of infections (18% vs. 25%), neoplasia (8 vs. 17%), and acute hepatitis (0 vs. 5%). Our subgroup analyses have shown that GF is highly effective in distinguishing AOSD from other IMIDs, but much more limited for some other etiological groups, such as hematologic malignancies or acute hepatitis. In the latter, a huge increase in ALT activity generally facilitates the differential diagnosis. The rheumatological recruitment for controls in the original publication may then explain the much lower number of false positives using the Fautrel criteria in this study [2]. In this diagnostic set, the absence of exclusion criteria may expose to decreased specificity and PPV (PPV 88.7% in the validation cohort [3] vs. 68.4% in ours, while the prevalence of AOSD was similar between the two series, 16% and 12% respectively).

Furthermore, our results indicated that, in the setting of hematological malignancies, a level of GF ≤ 20% was mostly suggestive of HLH. The literature on the value of GF in hematological malignancy is very scarce and does not allow for much speculation. Konijn et al. have shown that GF normalization was associated with clinical remission in hematological malignancies but the association with HLH was not mentioned [25]. Yet, considering our findings, the best recommendation is probably to alert the clinician who has to manage a patient with overlapping symptoms that the main (and almost only) differential diagnosis of AOSD flare is a hematological malignancy complicated by HLH. Javaux et al. have published different parameters that are useful for distinguishing HLH and AOSD flare, including lower fibrinogen, leukocyte and platelet counts, but no difference in GF fraction [7]. Another study showed that interleukin (IL)-18 levels have high accuracy for the differential diagnosis of AOSD and HLH but IL-18 is rarely available routinely [19].

For infectious diseases, we found that viral infections are associated with GF levels ≤ 20%, more frequently than bacterial infections and independently of HLH. This is of clinical importance as viral infections represent major differential diagnoses [26], especially for the monocyclic pattern of systemic AOSD. Therefore, a GF ≤ 20% is not an indicator to exclude viral infections. Nevertheless, we found no infectious disease associated with a GF ≤ 10%, and only three diagnoses under this threshold: HLH, AOSD, and acute hepatitis.

We propose a modified diagnostic score for AOSD, based on the Fautrel criteria with a GF threshold of 16%, that reduces the number of false positives, increases the specificity without losing any sensitivity, and increases the PPV by nearly 5 points. Of note, the threshold of GF ≤ 20% retained in the Fautrel criteria came from the preliminary study by Van Reeth et al. [23], and the evaluation study transformed it into a binary variable without prior evaluation of the best threshold for GF [2]. The limit of quantification of our assay was compatible with our 16% diagnostic threshold. If other teams wish to use this threshold, they must first ensure that the same applies in their laboratory.

The threshold of GF > 50% had an NPV of 100% in our series and may therefore be of interest to exclude AOSD, including for patients in remission. In addition, GF retained its diagnostic performances even if patients did not have elevated hyper-ferritinemia. We showed that GF remained significantly lower in AOSD in remission than in controls without active disease. This argues for the use of GF even after the AOSD flare has passed. Vignes et al. have already shown that, unlike TSF (which rapidly normalized with treatment and remission), GF remained low at three years [27]. Other markers such as free IL-18, soluble IL-2 receptor, or neutrophil-to-lymphocyte ratio have been evaluated for AOSD with good diagnostic accuracy or to monitor AOSD disease activity and therapeutic response [28,29,30,31]. Similar to GF, but unlike the other markers, free IL-18 remains abnormal in remission, even if its concentrations have been shown to be higher during the active phase of AOSD [28].

Our study showed that patients with decreased GF had a worse prognosis in terms of ICU admission and mortality within a month after the GF assay, independently of HLH. This prognostic value could not be validated in the subgroup analysis for AOSD due to the small sample size.

Using TSF-GF assays as an inclusion criterion, we identified cases and controls in a homogeneous and unselected population that provided a clinically relevant control group. The low prevalence of AOSD in our series may explain the relatively low PPV of our diagnostic sets. Nevertheless, this low prevalence is representative of the prevalence of AOSD in the general population.

A limitation of our study is its retrospective nature. To date, no diagnostic criteria or biomarkers have been formally validated and any prospective assessment of diagnostic criteria in the field of AOSD will remain very difficult because of its low incidence. An additional limitation of our study is the small number of cases compared with controls, seemingly due to the low prevalence of AOSD in our series. However, this low prevalence is representative of AOSD, even when evoked in a selected population with high pretest probability. This may explain why our cohort and that of Lebrun et al. have a similar order of magnitude in terms of AOSD frequency in the total patient population (12 vs. 16%, respectively) [3]. Another limitation is the identification of AOSD cases on the basis of clinician opinion. Yet no other approach would be entirely satisfactory in a condition for which there is no gold standard or consensus definition. This is why Yamaguchi [1] and Fautrel [2] also used expert judgement as a gold standard. Indeed, defining AOSD cases by the existing classification criteria would exclude all conflicting cases from the analysis and prevent the evaluation of those same previous sets of criteria.

## 5. Conclusions

GF is an accurate diagnostic test for AOSD, even in patients in remission or without elevated hyper-ferritinemia. The previously reported cut-off of 20% may lack specificity and could be improved by lowering it to 16%.

## Figures and Tables

**Table 1:** Main clinical and biological characteristics of patients with adult-onset Still’s disease (AOSD) and controls.

Characteristics (Unit)	AOSD *n* = 28	Controls *n* = 203	*p*-Value
Age (year)	41 (±18)	53 (±18)	0.001
BMI (kg/m^2^)	23 (±5)	24 (±5)	0.369
Male sex	12 (42.9%)	119 (58.6%)	0.154
Body temperature (°C)	39.1 (36.9–39.6)	37.9 (36.8–39.0)	0.017
Adenopathy	11 (39.3%)	30 (14.8%)	0.003
Hepatomegaly	4 (14.3%)	19 (9.4%)	0.496
Splenomegaly	4 (14.3%)	21 (10.3%)	0.518
Arthralgia	16 (57.1%)	56 (27.6%)	0.004
Arthritis	8 (28.6%)	31 (15.3%)	0.103
Pharyngitis	12 (42.9%)	16 (7.9%)	<0.001
Maculopapular rash	12 (42.9%)	31 (15.3%)	0.001
Transient erythema	6 (21.4%)	1 (0.5%)	<0.001
Glycosylated ferritin (%)	22.3 (±20.8)	39.3 (±17.6)	<0.001
Serum ferritin (µg/L)	1162 (336–3532)	717 (295–1942)	0.194
CRP (mg/L)	103 (19–177)	71 (19–155)	0.823
Fibrinogen levels (g/L)	5.3 (3.4–7.1)	4.7 (2.8–8.2)	0.77
ALT (UI/L)	51 (29–109)	34 (18–68)	0.016
AST (UI/L)	39 (28–85)	32 (20–67)	0.061
Total bilirubin (µmol/L)	7 (6–12)	8 (6–14)	0.243
Serum creatinine (µmol/L)	62 (54–75)	68 (56–81)	0.2
White Blood Cell Count (G/L)	9.8 (6.5–13.6)	7.9 (4.8–11.1)	0.038
Neutrophils (G/L)	7.3 (3.8–10.7)	4.5 (2.8–7.6)	0.012
Relative neutrophil count (%)	76 (63–82)	67 (55–77)	0.012
Lymphocytes (G/L)	1.6 (1.1–1.8)	1.3 (0.8–2.1)	0.89
Platelet (G/L)	241 (184–275)	242 (146–323)	0.886
Hemoglobin (g/L)	117 (100–131)	109 (88–134)	0.187
HLH	3 (10.7%)	22 (10.8%)	>0.999
Active disease	19 (67.9%)	183 (90.1%)	0.003

Abbreviations: AOSD, adult-onset Still’s disease; BMI, body mass index; CRP, C-reactive protein; ALT, alanine transaminase; AST, aspartate transaminase; HLH, hemophagocytic lymphohistiocytosis. Qualitative variables are *n* (%); quantitative variables are mean (±standard derivation) or median (first quartile-third quartile).

**Table 2:** The distribution of diagnoses in control group.

Diagnostic Category	Causes
**Immune-Mediated Inflammatory Diseases** 35% (*n* = 70)	**Idiopathic pericarditis** (*n* = 19)
	**Rheumatoid arthritis** (*n* = 13)
	**Miscellaneous systemic disorders** (*n* = 10): *TAFRO syndrome, inflammatory bowel disease, Sjögren’s syndrome, relapsing polychondritis, IgG4-related disease, autoimmune myositis*
	**Systemic lupus erythematosus** (*n* = 9)
	**Giant cell arteritis or polymyalgia rheumatica** (*n* = 6)
	**Spondyloarthropathies** (*n* = 5)
	**Vasculitis** (*n* = 5): *Polyarteritis nodosa, Urticarial vasculitis, Cryoglobulinemic vasculitis, Anti-neutrophil cytoplasmic autoantibody-associated vasculitis*
	**Autoinflammatory syndromes** (*n* = 3): *NLRP3 mutation, NLRC4 mutation, ROSAH syndrome*
**Infectious Diseases** 25 % (*n* = 51)	**Pyogenic bacteria** (*n* = 22): *Enterobacteriaceae, Staphylococcus, Streptococcus, Anaerobic*
	**Viral infection** (*n* = 21): *Human herpesvirus (EBV, CMV), Influenza, Parvovirus B19, human immunodeficiency viruses (HIV)*
	**Intracellular bacteria** (*n* = 7): *Tuberculosis, Coxiella burnetii, Tropheryma whipplei, Legionella*
	**Parasitic disease** (*n* = 1): *Blastocystis hominis*
**Hematologic Malignancies** 12% (*n* = 24)	**Lymphoid malignancies** (*n* = 15): *Hodgkin lymphoma, B cell non-Hodgkin lymphoma, T cell non-Hodgkin lymphoma, multiple myeloma*
	**Myeloid malignancies** (*n* = 9): *acute leukemia, myelodysplastic syndrome, VEXAS syndrome, mastocytosis*
**Solid Cancers** 5% (*n* = 11)	**Metastatic cancer** (*n* = 7)
	**Localized cancer** (*n* = 4)
**Acute Hepatitis** 5% (*n* = 11)	**Drug induced hepatitis** (*n* = 5)
	**Autoimmune hepatitis** (*n* = 3)
	**Viral hepatitis** (*n* = 3)
**Other** 18% (*n* = 36)	**Crystal arthropathies** (*n* = 5)
	**Urticaria** (*n* = 8)
	**Various** (*n* = 23): *DRESS syndrome, Osteoarthritis, Dressler syndrome, Thrombotic microangiopathy, Venous Thromboembolic Disease, Fever of unknown origin, Fibromyalgia, Focal and segmental glomerulosclerosis, Myocardial infarction, subacute granulomatous thyroiditis, Alcohol use disorder*

Abbreviations: NLRP3, NOD-like receptor family, pyrin domain containing protein 3; NLRC4, NOD-like receptor family CARD domain containing protein 4; EBV, Epstein–Barr virus; CMV, cytomegalovirus; VEXAS syndrome, Vacuoles E1-enzyme X-linked Autoinflammatory and Somatic syndrome; ROSAH syndrome, Retinal dystrophy Optic nerve edema Splenomegaly Anhidrosis and migraine Headache syndrome; TAFRO syndrome, Thrombocytopenia Anasarca reticulin Fibrosis Renal insufficiency and Organomegaly syndrome (idiopathic multicentric Castleman disease subtype); Abbreviations need to be redefined in the first time they are used in both figures and tables. Drug reaction with eosinophilia and systemic symptoms.

**Table 3:** Diagnostic value of glycosylated ferritin and classification criteria in adult-onset Still’s disease.

Diagnostic Criteria	Specificity	Sensitivity	NPV	PPV
GF ≤ 20%	83.6%	63.2%	95.6%	28.6%
GF ≤ 16%	88.5%	63.2%	95.9%	36.4%
GF ≤ 10%	95.6%	52.6%	95.1%	55.6%
Fautrel	96.7%	68.4%	96.7%	68.4%
Fautrel-16	97.3%	68.4%	96.7%	72.2%
Yamaguchi	99.4%	68.4%	96.8%	92.9%
GF ≤ 50%	23.0%	100.0%	100.0%	11.9%

Abbreviations: GF, glycosylated ferritin; NPV, negative predictive value; PPV, positive predictive value.

## Data Availability

Additional data may be obtained on reasonable request from the corresponding author.

## Supplementary Materials

The following supporting information can be downloaded at: https://www.mdpi.com/article/10.3390/jcm11175012/s1, Figure S1: Survival probability without death or intensive care unit admission according to glycosylated ferritin (GF) level, Figure S2: Hemophagocytic lympho-histiocytosis prevalence in hematologic malignancies (*n* = 24) according to glycosylated ferritin (GF) level.
