# Supplementary material for: Evaluation of Glycosylated Ferritin in Adult-Onset Still’s Disease and Differential Diagnoses
Source: J Clin Med. 2022 Aug 26;11(17):5012. doi: 10.3390/jcm11175012 (PMC9456550; doi:10.3390/jcm11175012)

Supplementary Material

Article: Evaluation of glycosylated ferritin in adult-onset Still's disease and differential diagnoses, by Arthur Guerber et al.

Supplemental Figure S1. Survival probability without death or intensive care unit admission according to glycosylated ferritin (GF) level

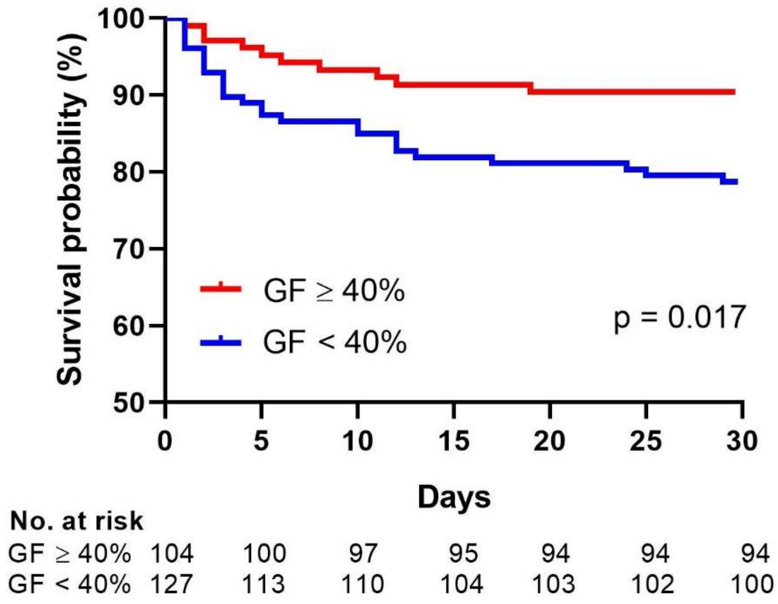

Supplemental Figure S2. Hemophagocytic lymphohistiocytosis prevalence in hematologic malignancies (n=24) according to glycosylated ferritin (GF) level

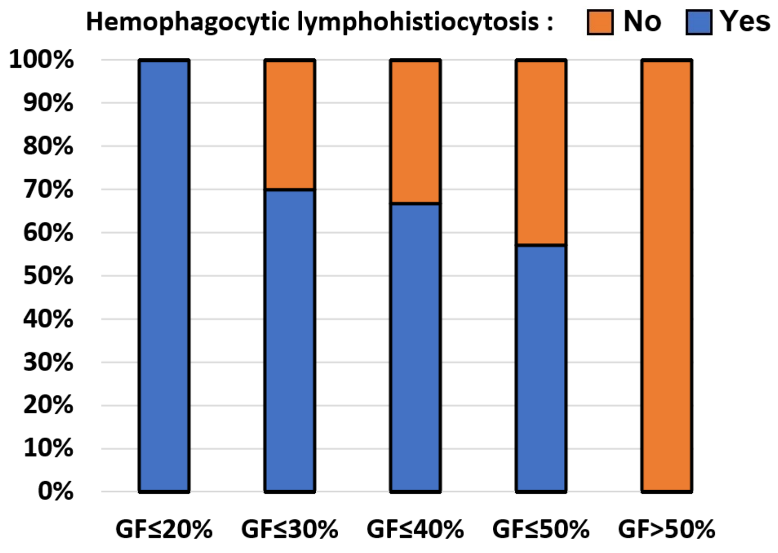

Supplement: Supplementary file 1 [file jcm-11-05012-s001.zip › jcm-1844539-supplementary.pdf]
